# Statistical Evaluation of Waveform Collapse Reveals Scale-Free Properties of Neuronal Avalanches

**DOI:** 10.3389/fncom.2016.00029

**Published:** 2016-04-07

**Authors:** Aleena Shaukat, Jean-Philippe Thivierge

**Affiliations:** ^1^School of Psychology, University of OttawaOttawa, ON, Canada; ^2^Center for Neural Dynamics, University of OttawaOttawa, ON, Canada

**Keywords:** neuronal avalanches, *in vitro*, bursts, network dynamics, cultured neuronal networks, multi-electrode array, criticality

## Abstract

Neural avalanches are a prominent form of brain activity characterized by network-wide bursts whose statistics follow a power-law distribution with a slope near 3/2. Recent work suggests that avalanches of different durations can be rescaled and thus collapsed together. This collapse mirrors work in statistical physics where it is proposed to form a signature of systems evolving in a critical state. However, no rigorous statistical test has been proposed to examine the degree to which neuronal avalanches collapse together. Here, we describe a statistical test based on functional data analysis, where raw avalanches are first smoothed with a Fourier basis, then rescaled using a time-warping function. Finally, an F ratio test combined with a bootstrap permutation is employed to determine if avalanches collapse together in a statistically reliable fashion. To illustrate this approach, we recorded avalanches from cortical cultures on multielectrode arrays as in previous work. Analyses show that avalanches of various durations can be collapsed together in a statistically robust fashion. However, a principal components analysis revealed that the offset of avalanches resulted in marked variance in the time-warping function, thus arguing for limitations to the strict fractal nature of avalanche dynamics. We compared these results with those obtained from cultures treated with an AMPA/NMDA receptor antagonist (APV/DNQX), which yield a power-law of avalanche durations with a slope greater than 3/2. When collapsed together, these avalanches showed marked misalignments both at onset and offset time-points. In sum, the proposed statistical evaluation suggests the presence of scale-free avalanche waveforms and constitutes an avenue for examining critical dynamics in neuronal systems.

## Introduction

Neural avalanches are a form of brain dynamics observed both *in vivo* and *in vitro* and characterized by bursts of activity whose statistics follow a power-law distribution (Plenz and Thiagarajan, 2007). Several physical systems that evolve in a critical state exhibit power-law scaling (Bak et al., 1987). Whether such power-law scaling reflects criticality in neuronal recordings, however, remains debated (Touboul and Destexhe, 2010; Klaus et al., 2011; Beggs and Timme, 2012). This debate is fueled by the presence of power-law scaling in stochastic systems that are not in a critical state (Benayoun et al., 2010). Addressing this question has fundamental implications in neural systems, as theoretical work associates the critical state with optimal information processing, optimal storage, and flexible responses (Beggs and Plenz, 2003; Shew et al., 2009, 2011).

One aspect of physical systems that weighs in favor of criticality is the presence of fractal relations at different times scales (Sethna et al., 2001). Preliminary work has tested this idea by taking avalanches of different durations, and rescaling the amplitude and timescale of their mean activity over time (Friedman et al., 2012). As a result, avalanches of different durations show a strikingly similar profile of activation. This scaling property suggests the presence of similar avalanche dynamics across a range of temporal scales.

However, no statistical test has been proposed to examine the reliability of this temporal scaling. Such test is not only necessary to quantify this phenomenon under normal recordings, but also its potential breakdown in neural activity altered by pharmacology (Vincent et al., 2013).

Here, we address this question with a technique of functional data analysis applied to recordings of cultured cortical neurons plated on multielectrode arrays (Ramsay and Silverman, 2005). The aim of this technique is to transform a set of data into smooth differentiable functions. This is done by approximating the data by a weighted sum of basis functions. The resulting functions can then be rescaled using a time-warping function, and analyzed using a generalized linear model (GLM).

This paper is structured as follows. First, we describe some basic aspects of neuronal avalanches, including scale-free distributions of time durations and counts of active neurons. Second, we describe how data can be collapsed using a time-warping function that rescales avalanches using their total duration and mean amplitude. Third, we test the statistical significance of the data collapse by combining a GLM and permutation test. Finally, we examine the time-warping function using a principal components analysis. We compare data collapse obtained from control and pharmacologically altered activity under APV/DNQX, an AMPA/NMDA receptor antagonist known to disrupt avalanches by markedly increasing the slope of the best-fitting power-law describing their distribution (Vincent et al., 2012). Results show conditions that lead to a statistically robust collapse of avalanches and carry implications for the investigation of criticality in neuronal systems.

## Methods

Experiments were performed as previously described following approval from the Human Health Therapeutics Animal Care Committee at the National Research Council Canada. Cultures were inspected to ensure that neurons exhibited a dense homogeneous monolayer. Recordings were performed between 14 and 19 days *in vitro*, an age when cultures have sufficiently matured to produce maximal firing rates and most channels are active (Tauskela et al., 2008; Vincent et al., 2013). Pharmacological agents were added directly to the medium of cultures (AVP/DNQX = (2R)-amino-5-phosphonovaleric acid + 6,7-dinitroquinoxaline-2,3-dione). Drugs were prepared from stock solutions, with final bath concentrations of 2 μM DNQX, and 20 μM APV. A 10 min wash-in period preceded all recordings.

Recordings were performed at 37°C with 64-channel multielectrode arrays using MCRack software (Multi Channel Systems, Reutlingen, Germany). All recordings were carried out for 20 min duration. Signals were acquired at 5 kHz, then downsampled to 1 kHz and high-pass filtered using a cut-off frequency of 200 Hz. Online extracellular spike detect was performed using MCS software.

All offline analyses were performed with custom scripts in Matlab (Mathworks, Natick, MA). Neural avalanches were identified using non-overlapping time bins of fixed size (10 ms). An avalanche was defined as a series of consecutive bins where all bins have at least one spike. An avalanche must be preceded and followed by at least one time bin without spikes. Source code on functional data analysis is freely available online (http://www.functionaldata.org).

## Results

### Neuronal avalanches

**Figure 1:**
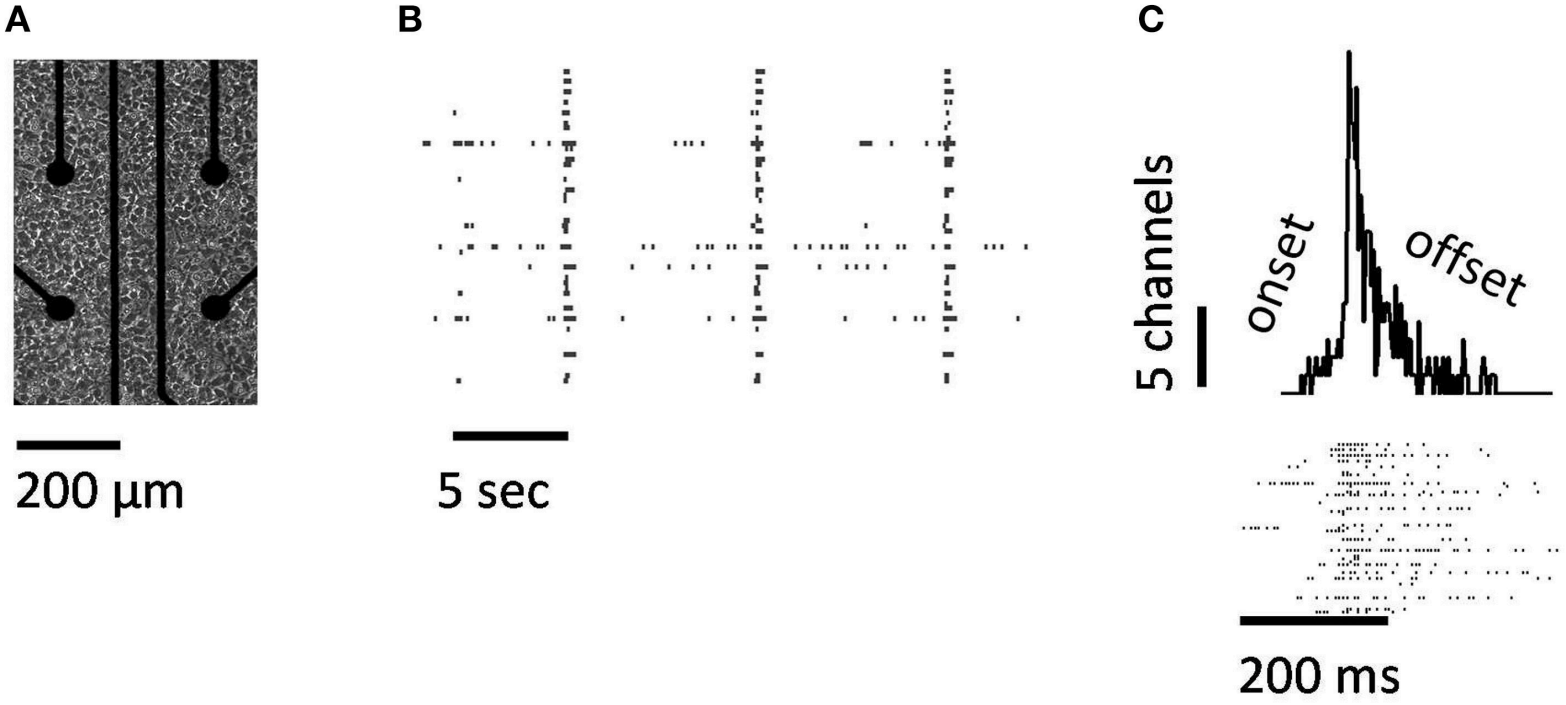
**Neuronal avalanches obtained from cortical cultures plated on multielectrode arrays**. **(A)** Cortical neurons on multielectrode array. Only 4 of the 64 electrodes are shown. **(B)** Spike raster obtained from a control culture. **(C)** A single avalanche, showing spike raster (bottom panel) and peri-stimulus time histogram relative to avalanche onset (top panel).

**Figure 2:**
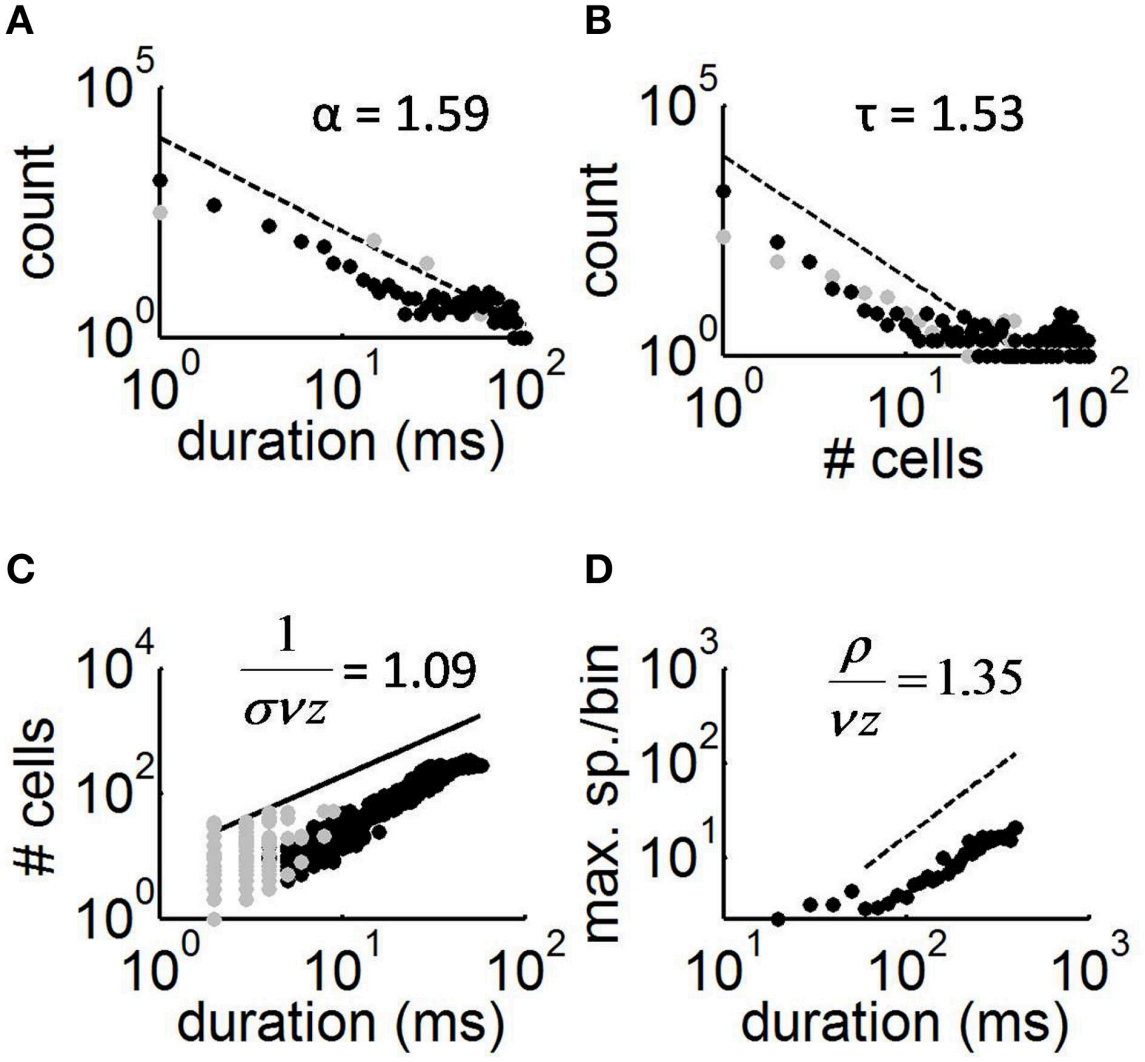
**Avalanche distributions and scaling relations**. **(A)** Distribution of avalanche duration (black dots) and best-fitting slope of power-law obtained by maximum likelihood estimation (Langlois et al., 2014). Gray dots, distribution obtained in cultures treated with APV/DNQX. **(B)** Distribution of the number of cells activated per avalanche. **(C)** Relation between the duration of avalanches and cell count. **(D)** Relation between the maximum spikes per time-bin in average avalanches of different durations vs. the duration of avalanches.

A total of 6 control cultures were analyzed (see Figure 1A for an illustration of multielectrode array). These data exhibited bursts of activity characteristic of neuronal avalanches (Figures 1B,C). A full analysis of avalanches for these data is available elsewhere, and shows that the duration of avalanches follows a power-law with slope close to 3/2 (Figure 2A; Vincent et al., 2012; Thivierge, 2014). A rigorous maximum likelihood method was employed to show that a power-law offered a closer fit than an exponential function (Langlois et al., 2014).

Individual avalanches were characterized by a sharp increase in neural activity between the onset and peak amplitude, followed by a more gradual offset (Figure 1C). The mean rate of increase in neural activity from the onset of an avalanche to its maximal amplitude was 1.28 spikes/ms (s.d. 0.16), which is close to both experimental (Eytan and Marom, 2006) and theoretical (Thivierge and Cisek, 2008) reports. The total duration of avalanches varied between 20 and 580 ms (mean of 40.89, s.d. of 8.03). Both the duration of avalanches and the number of cells activated followed a power-law distribution (Figures 2A,B), with scaling exponents α = 1.59 (s.d. 0.17) and τ = 1.53 (s.d. 0.09), respectively (Table 1). In turn, avalanche durations and cell count were related to each other by a power-law with exponent 1.09 (s.d. 0.004) (Figure 2C). In keeping with previous work, we refer to the latter exponent using the notation 1∕σ*νz* (Mehta et al., 2002). These exponents are slightly outside those predicted by mean field values (τ = 3∕2, α = 2.0, and 1∕σ*νz* = 2.0). In spite of this finding, the exponent relation for critical systems

(1) $$\begin{array}{l} \frac{\alpha -1}{\tau -1}=\frac{1}{\sigma \nu z} \end{array}$$

is approximated here, with 1/σ*νz* being slightly overestimated (the lefthand side of Equation 1 yields 1.11).

**Table 1:** **Summary of scaling exponents and mean-field theory**.

**Description**	**Exponent**	**Mean-field theory**
Duration distribution	α = 1.59 (s.d. 0.17)	α = 2.0
Size distribution (# of cells)	τ = 1.53 (s.d. 0.09)	τ = 1.5
Duration vs. size	1/σ*νz* = 1.09 (s.d. 0.004)	1/σ*νz* = 2.0
Max. spikes per time-bin	μ = 1.44 (s.d. 0.12)	μ = 2

An alternative means of estimating the above exponents is to consider the maximum number of spikes generated during a given time-bin of avalanches. For this analysis, we first used bins of 25 ms to average avalanches of similar duration together. In this way, an individual mean was obtained for avalanches between 20 and 45 ms, 45 and 70 ms, and so on until we reached the maximum avalanche duration of 580 ms. The distribution of maximum spikes per time-bin for these mean avalanches followed a power-law with exponent μ = 1.44 (s.d. 0.12). The relation between this exponent and α = 1.59 (obtained from the duration of avalanches) is described by the exponent ρ∕*vz* (LeBlanc et al., 2013). From mean-field theory, we expect the following relation

(2) $$\begin{array}{l} \frac{\alpha -1}{\mu -1}=\frac{\rho }{vz}, \end{array}$$

to be approximated here, with the value (α − 1)∕(μ − 1) = 1.34 being close to ρ∕*vz* = 1.35 (Figure 2D).

In previous work, the exponents α, τ, and 1∕σ*νz* are combined with a universal scaling function (a set of orthonormal polynomials) to examine whether avalanches of different duration can be collapsed together (Sethna et al., 2001; Friedman et al., 2012). Here, however, we take a different approach that offers a statistical criterion for testing the collapse of avalanches, as described next.

### Avalanche collapse

We employed a technique termed functional data analysis to examine the degree to which mean avalanches of various durations could be rescaled and collapsed onto each other. First, mean avalanches were filtered with a 2nd order low-pass Butterworth filter with a cut-off frequency of 100 Hz. Then, we represented mean avalanches of a given duration by a continuous variable *x*_*j*_ corresponding to the spike rate over time, sampled at 1 kHz. Each mean avalanche was smoothed by a linear mixture of Fourier basis ϕ_*k*_, where *k* = 1,…,*K*,

(3) $$\begin{array}{l} y\left(t\right)=\overset{K}{\underset{k=1}{\sum }}c_{k}\phi_{k}\left(t\right), \end{array}$$

given *c*_*k*_ coefficients and *K* number of basis functions defined by ϕ_0_(*t*) = 1, ϕ_2*r*−1_(*t*) = sin*rωt*, and ϕ_2*r*_(*t*) = cos*rωt*, where *t* indexes time within the interval *T* corresponding to the length of a given mean avalanche. The parameter ω defines the period 2πω and is set such that this period is equal to *T*. By allowing the *n* × *K* matrix Φ = {ϕ_*k*_(*t*_*j*_)} of basis functions to be of full rank, we obtained a close approximation *y*(*t*_*j*_) ≈ *x*_*j*_ for each *j* when *K* = *n* by fitting the coefficients *c*_*k*_. These coefficients were adjusted to minimize a sum of squared errors between data points *x*_*j*_ and basis Φ

(4) $$\begin{array}{l} SSE\left(x|c\right)={\left(x-\Phi c\right)}^{\prime }\left(x-\Phi c\right), \end{array}$$

where the vector *c* contains the coefficients *c*_*k*_. To avoid overfitting the data, we added a regularization term to Equation (4) that penalizes the second order derivative of *y*(*t*),

(5) $$\begin{array}{l} R\left(y\right)=\int {\left[\partial^{2}y\left(t\right)∕{\left(\partial t\right)}^{2}\right]}^{2}dt, \end{array}$$

thus yielding

(6) $$\begin{array}{l} SSE_{\lambda }\left(x|c\right)={\left(x-\Phi c\right)}^{\prime }\left(x-\Phi c\right)+\lambda R\left(y\right), \end{array}$$

where λ = 0.001 controls the amount of regularization applied. The target precision for Equation (6) is 0.0001. Equation (6) can be optimized in linear *O*(*n*) time using a least squares method described elsewhere in full detail (Green and Silverman, 1994). One assumption is that the noise is of higher frequency than the signal. This is justified because neuronal avalanches represent population-wide fluctuations in signals and have a time-course that is much slower than high-frequency “jitter” induced by single spikes.

**Figure 3:**
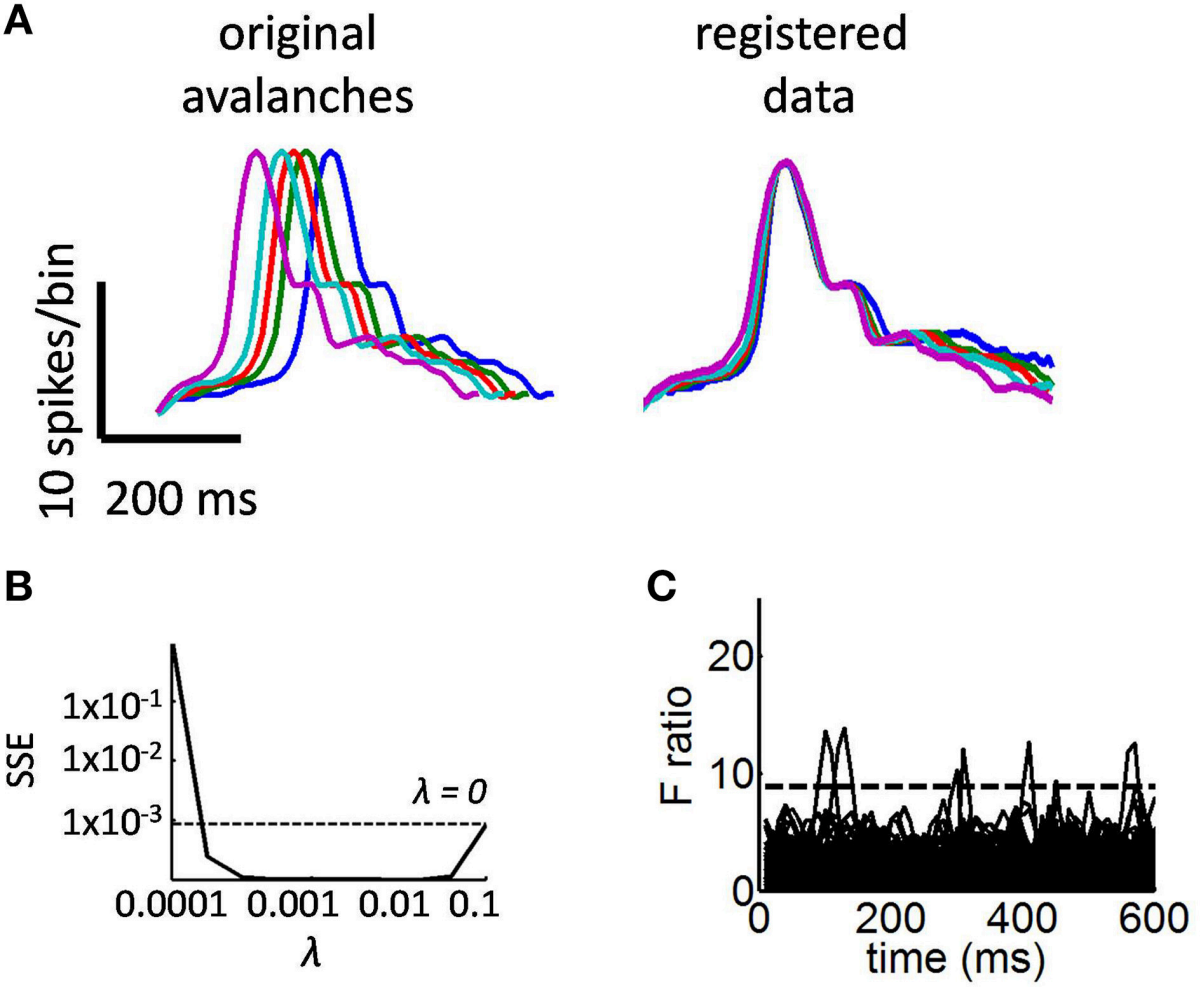
**The shape of mean avalanches of different durations shows a statistically reliable collapse**. **(A)** Left panel: example of avalanches of different durations. These avalanches were smoothed using a Fourier basis (Equation 3). Right panel: avalanches registered using a time-warping function (Equation 7). Avalanches are rescaled by their maximum amplitude for ease of visualization. **(B)** Sum of squared errors (SSE) obtained from Equation (6) as a function of the regularization parameter λ. **(C)** F ratio obtained from a functional linear model. Dashed line, critical *F*-value. Each of 1000 bootstrap permutations is displayed by a solid black line.

Examples of smoothed avalanches are shown in Figure 3A (left panel). To confirm that our choice of parameter λ in Equation (6) yielded low SSE, we tested a range of values between λ = 0.001 and λ = 0.1 (Figure 3B). Despite the smoothed avalanches having different durations, their overall shape was strikingly similar. It may thus be possible to rescale these avalanches in time such that their activity aligns with each other. This was achieved by computing a time-warping function for each mean avalanche. An advantage of this approach is that it allows us to devise a statistical analysis based on principal components analysis and GLM to examine the degree to which avalanches collapse together (see below).

The time-warping transformation of *y*(*t*) is defined as

(7) $$\begin{array}{l} y^{*}\left(t\right)=y_{i}\left[h_{i}\left(t\right)\right], \end{array}$$

given the time-warping function *h*_*i*_ (*t*), where *i* indexes individual avalanches. Here, *h* implements a transformation

(8) $$\begin{array}{l} h_{i}\left(t\right)=\Delta \left(D^{-1}\exp D^{-1}w\right)\left(t\right), \end{array}$$

where

(9) $$\begin{array}{l} \Delta =T∕\left[D^{-1}\exp D^{-1}w\left(t\right)\right] \end{array}$$

and *D*^−1^ is an integration operator with lower limit of zero. These time-warping functions are fitted with a least-squares criterion using a Newton-Raphson algorithm as described elsewhere (Ramsay and Silverman, 2005).

The above warping functions allowed for avalanches to be collapsed onto each other (Figure 2A, right panel). The collapse of avalanches was particularly accurate around the peak of avalanches, but less so toward the offset. To evaluate the degree to which avalanches collapsed together, we employed a combination of GLM and bootstrap permutation. This procedure allowed us to identify time-points where avalanches collapsed in a statistically robust manner, as described next.

### Generalized linear modeling

First, we randomly divided all mean avalanches of various durations into two surrogate groups of equal size. Then, we fitted the resulting data set using the linear model

(10) $$\begin{array}{l} y_{kg}\left(t\right)=\mu \left(t\right)+\alpha_{g}\left(t\right)+\varepsilon_{kg}\left(t\right), \end{array}$$

where μ(*t*) is the grand mean of the data (i.e., the average avalanche shape over all groups), α_*g*_(*t*) is the deviation of the average avalanche shape in a given surrogate group from the grand mean, and ε_*kg*_(*t*) is the residual deviation of the *k*th avalanche in group *g* from the mean avalanche profile. We constrained α_*g*_(*t*) such that $\underset{g}{\sum }\alpha_{g}\left(t\right)=0$ for all *t*. This constraint is necessary to insure that α_*g*_(*t*) can be identified uniquely as belonging to a given surrogate group. To fit this model, we designed a matrix **Z** of size *N* (avalanche) × *G*+1 (surrogate groups). We indexed the rows and columns of **Z** using (*k*,*g*), corresponding to mean avalanche *k* in group *g*. Row *g* of matrix **Z** had a one in the first column, a one in column *g* + 1, and zeroes elsewhere. For instance, with *N* = 4 avalanches and *G* = 2 groups, and assuming that the first two rows are assigned to group 1 and the remaining 2 rows are assigned to group 2, the **Z** matrix would be

$$\begin{array}{l} Z=\left[\begin{array}{ccc} 1 & 1 & 0\\ 1 & 1 & 0\\ 1 & 0 & 1\\ 1 & 0 & 1 \end{array}\right]. \end{array}$$

The linear model of Equation (10) can then be rewritten as

(11) $$\begin{array}{l} y\left(t\right)=\overset{N}{\underset{j=1}{\sum }}z_{\left(k,g\right)j}\varphi_{j}\left(t\right)+\varepsilon_{kg}\left(t\right), \end{array}$$

where φ_*j*_(*t*) are a set of *N* regression functions, φ_*j*_, φ_1_ = μ, φ_2_ = α_1_, and so on, up to φ_*N*_ = α_*N*−1_, yielding the vector φ = [μ, α_1_, α_2_, …, α_*N*_]. This model can be fitted with a least squares criterion,

(12) $$\begin{array}{l} SSE\left(\varphi \right)=\underset{g}{\sum }\underset{k}{\sum }\int {\left[y_{kg}\left(t\right)-{\left(Z\varphi \right)}_{kg}\right]}^{2}dt, \end{array}$$

where

$$\begin{array}{l} {\left(Z\varphi \right)}_{kg}=\overset{N}{\underset{j=1}{\sum }}z_{\left(k,g\right)}\varphi_{j}\left(t\right), \end{array}$$

minimized subject to constraint $\overset{N}{\underset{j=1}{\sum }}\varphi_{j}=\mu$. We assessed the fit of Equation (11) at each time point *t* using

(13) $$\begin{array}{l} SSE\left(t\right)=\underset{k,g}{\sum }{\left[y_{kg}\left(t\right)-{\left(Z\hat{\varphi }\right)}_{kg}\left(t\right)\right]}^{2} \end{array}$$

evaluated at the fitted values of φ obtained by minimizing (Equation 12). We then tested for statistical robustness using an F ratio,

(14) $$\begin{array}{l} F_{ratio}\left(t\right)=\frac{\begin{array}{c} \underset{k,g}{\sum }{\left[y_{kg}\left(t\right)-\hat{\mu }\left(t\right)\right]}^{2}\\ \;-\underset{k,g}{\sum }{\left[y_{kg}\left(t\right)-{\left(Z\hat{\varphi }\right)}_{kg}\left(t\right)\right]}^{2}∕df_{model} \end{array}}{\underset{k,g}{\sum }{\left[y_{kg}\left(t\right)-{\left(Z\hat{\varphi }\right)}_{kg}\left(t\right)\right]}^{2}∕df_{error}} \end{array}$$

where *df*_*model*_ = *G*−1 and *df*_*error*_ = *N*−2 are the degrees of freedom associated with the linear model and error, respectively. Values of *F*_*ratio*_(*t*) obtained by Equation (14) were compared to a critical value from the F distribution. We repeated the above procedure (Equations 10–14) 1000 times, each time using a random assignment of avalanches to each of the two surrogate groups in Equation (10). Finally, we identified time-points of *F*_*ratio*_(*t*) where >1% of all 1000 random permutations were above the critical *F*-value in Equation (14), corresponding to points where data did not collapse reliably.

**Figure 4:**
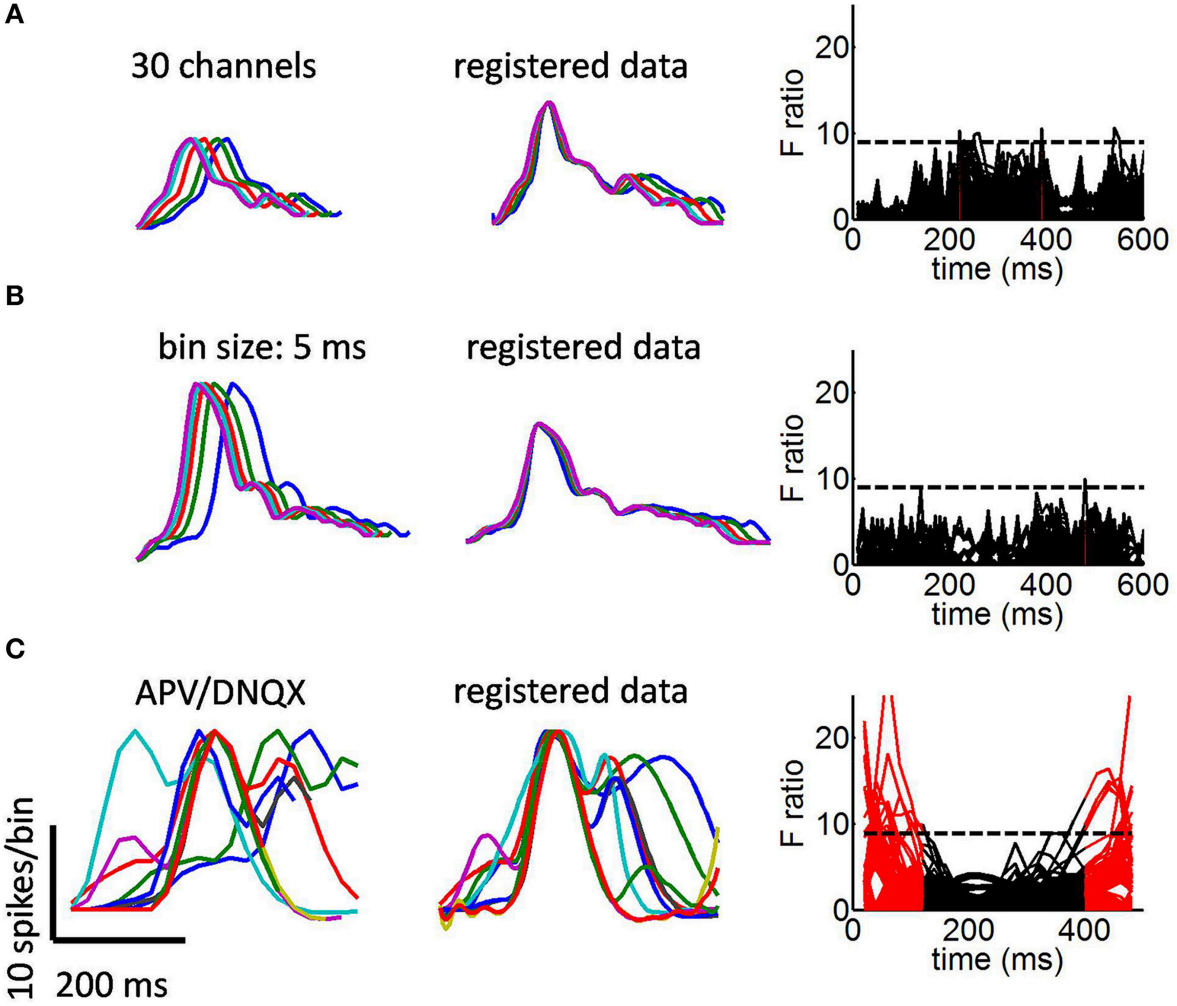
**Avalanche collapse under various conditions**. **(A)** Avalanche collapse with a random subsample of 30 channels. **(B)** Detecting avalanches with a bin size of 5 ms (instead of the 10 ms default). **(C)** Avalanches in cultures treated with APV/DNQX. Times when F ratio exceeded the critical value in at least 1% of all bootstrap realizations are shown in red.

Using the above procedure, we found that despite slight misalignments at the offset of avalanches, all time points were collapsed above chance levels (Figure 3C; time-points that are robustly aligned are shown by solid black lines). Similar results are found when considering only a subset of 30 channels (Figure 4A) and when altering the duration of time bins for detecting avalanches (Figure 4B, see Methods).

We compared the above results with those of cultures treated with APV/DNQX. Distributions of avalanche duration, cell count, as well as the relation between these measures are shown in Figures 2A-C, respectively. In previous work, we showed that APV/DNQX cultures yield avalanches with power-laws greater than 3/2 (Vincent et al., 2012). Smoothed mean avalanches recorded in this condition displayed a variety of shapes (Figure 4C, left panel). Attempts to collapse these shapes together resulted in marked discrepancies both before and after the peak of avalanches (Figure 4C, middle panel). These discrepancies were reflected in an F ratio test (Figure 4C, right panel). These results differ sharply from those of control cultures, which displayed a robust collapse at all time-points of avalanches. Importantly, while the poor collapse of APV/DNQX data may be anticipated by observation of the original avalanche shapes (Figure 4C, left panel), results of the F ratio test provide a benchmark for the adequate behavior of the proposed framework.

In sum, the above analysis shows that control cultures generate avalanches across a range of different durations. These avalanches show a striking similarity as highlighted by a time-warping function that robustly collapsed them together. By comparison, cultures treated with APV/DNQX, an AMPA/NMDA antagonist that disrupts the scale-free distribution of avalanches, yielded activity that could not be collapsed to the same degree.

### Principal components analysis

**Figure 5:**
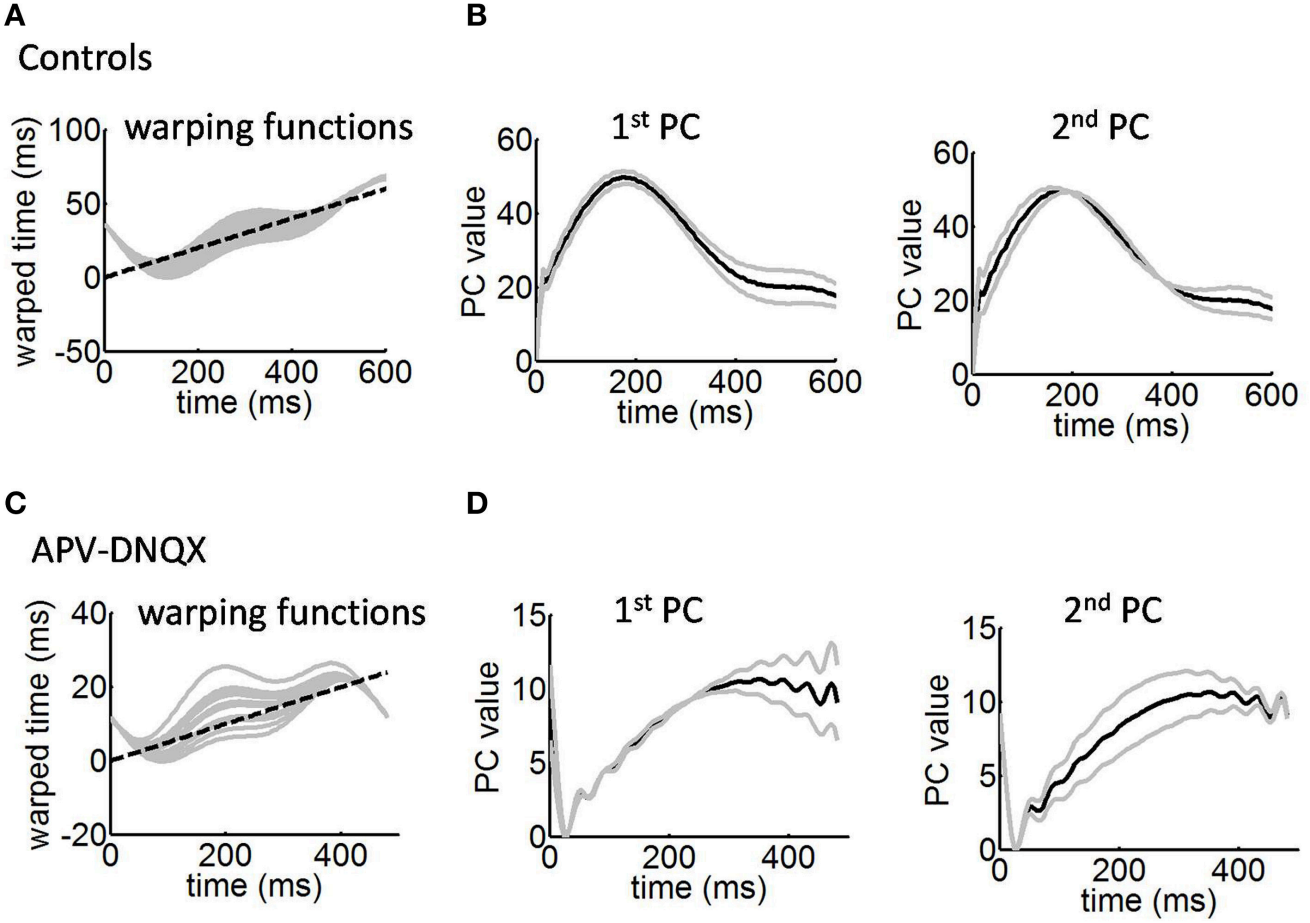
**Warping functions employed to collapse the avalanches**. **(A)** Solid gray lines, examples of warping functions for mean avalanches of different durations. Dashed black line is a line of unity. **(B)** Largest two principal components (PCs) of the warping functions from **(A)**. Solid gray lines, perturbations around the mean induced by each principal component (Equations 19–20). Solid black line, mean time-warping function of avalanches over time. **(C)** Warping functions for mean avalanches under APV/DNQX. **(D)** Largest two principal components of the warping functions in **(C)**.

While the above results are consistent with the idea that avalanches in a critical state of activity can be reliably collapsed together (Sethna et al., 2001; Friedman et al., 2012), they fail to characterize the linear time-warping transformation that makes this possible. Examples of time-warping functions for mean avalanches of different durations are shown in Figure 5A. Departures from a line of unity (Figure 5A, dashed line) are observed predominantly at the onset and offset of avalanches. To further examine this effect, we entered the time-warping functions *h*_*i*_(*t*) in a principal components analysis (PCA). This PCA performs a linear approximation of the time-warping functions as follows:

(15) $$\begin{array}{l} {ĥ}_{i}\left(t\right)=\overset{K}{\underset{k=1}{\sum }}f_{ik}\xi_{k}\left(t\right), \end{array}$$

where ξ_*k*_ are a set of orthonormal functions and

(16) $$\begin{array}{l} f_{ik}=\int_{0}^{T}x_{i}\left(t\right)\xi_{k}\left(t\right)dt. \end{array}$$

The fitting criterion for Equation (15) is an integrated sum of squared errors,

(17) $$\begin{array}{l} SSE=\overset{N}{\underset{i=1}{\sum }}||h_{i}-{ĥ}_{i}|{|}^{2}, \end{array}$$

where ||·|| is a norm operator. To minimize the above criterion, we sought a set of functions ξ_*k*_ by finding the solution with largest eigenvalue *e* of the eigenvector problem,

(18) $$\begin{array}{l} V\xi =e\xi , \end{array}$$

where **V** is the variance-covariance matrix of the time-warping functions *h*_*i*_(*t*). In order to visualize the results of the PCA, we plotted the mean time-warping function, plus or minus a multiple of the PCA function. To choose an adequate multiple to use, we defined a constant *C* to be the root-mean-square difference between the estimated mean function ($\hat{\mu }$) and the overall time average ($\bar{\mu }$):

(19) $$\begin{array}{l} C^{2}=T^{-1}||\hat{\mu }-\bar{\mu }|{|}^{2}, \end{array}$$

where

(20) $$\begin{array}{l} \bar{\mu }=T^{-1}\int \hat{\mu }\left(t\right)dt. \end{array}$$

We then plotted $\hat{\mu }$ and $\hat{\mu }\pm 0.2C{ĥ}_{i}$, where the constant 0.2 gives results that are easy to interpret.

In control cultures, the largest principal component shows stronger values toward the offset of the time-warping function (Figure 5B, left panel). This is consistent with time-warping being more variable in that time window, because avalanche collapse is weaker (Figure 4A, middle panel). The second largest principal component shows divergence from the mean both at the onset and offset of avalanches (Figure 5B, right panel). Together, the two largest principal components account for 93% of variance across time-warping functions. We compared these results with those obtained from time-warping functions for the APV/DNQX data (Figure 5C). As with controls, the largest principal component was associated with the offset of avalanches (Figure 5D, left panel), while the second largest principal component covered most of the avalanche duration (Figure 5D, right panel).

In sum, analyses of the time-warping functions reveal that misalignments arise most strongly toward the offset of avalanches, with slight misalignments also arising between the onset and peak of avalanches.

## Discussion

In this paper, we examined whether neuronal avalanches of different durations can be collapsed together (Sethna et al., 2001; Friedman et al., 2012). One implication of avalanche collapse is that they form a key signature of criticality in neuronal systems, as they reflect a fractal property of activity at different timescales (Beggs and Timme, 2012). We tested this idea using a statistical evaluation of avalanche collapse based on functional data analysis (Ramsay and Silverman, 2005). Our results show that avalanches could be collapsed together in a statistically robust fashion. By comparison, recordings under APV/DNQX, which alter the scale-free distribution of avalanches, yielded an unreliable collapse of avalanches at both the onset and offset time-points.

These results thus support the collapse of neuronal avalanches and highlight experimental conditions under which it may be disrupted. A follow-up examination of the time-warping function employed to collapse avalanches, however, showed variations across avalanches, particularly near their offset, thus calling into question the strict fractal nature of this activity.

The proposed framework for evaluating avalanche collapse may serve to discern amongst different classes of models that generate power-law distributions of avalanches (Levina et al., 2007; Thivierge and Cisek, 2008; Benayoun et al., 2010; Rubinov et al., 2011). While these models may successfully reproduce power-law statistics of avalanches observed experimentally, it is unclear whether they would also capture avalanche collapse. One crucial factor in generating avalanche collapse may be the connectivity of neural models (Friedman et al., 2012). A full examination of this hypothesis, however, remains to be performed. Further work is also required to examine the collapse of neuronal avalanches in other datasets where power-law distributions have been reported, including *in vivo* activity (Petermann et al., 2009).

While the framework described here relies on functional data analysis, alternatives are possible, including the use of dynamic time warping (Theodoridis and Koutroumbas, 2009). As with functional data analysis, dynamic time warping uses a time transformation to compare time-series that vary in speed. However, a disadvantage of dynamic time warping is that it does not guarantee that the warped time-series will result in smoothly differentiable functions, which prevents further analyses such as the identification of peaks (Thivierge, 2009) as well as a functional PCA (Figure 5).

It remains unclear whether the statistical collapse of avalanches provides a definitive signature of critical dynamics in neural systems. Crucially, stochastic systems have been shown to capture power-law distributions of avalanches (Touboul and Destexhe, 2010). It remains to be shown whether such systems also exhibit an avalanche collapse in conditions that mimic normal and pharmacologically-altered states. Analyses of such systems could test a further prediction of critical phenomena not addressed here, namely that the size distribution of avalanches should scale as

(21) $$\begin{array}{l} s^{-\tau }L\left(s{\left(b-d\right)}^{1∕\sigma }\right) \end{array}$$

where *L*(·) is a universal scaling function and *b* is a parameter tuned away from the critical value *d*.

One limitation of the approach proposed here is that we are constrained to collapsing avalanche shapes along the time axis, and not along their amplitudes. Thus, the more general problem of data collapse remained to be addressed. However, our current work offers an important step in this direction by proposing a statistical framework to evaluate the quality of scaling collapse in avalanche data, and may be expanded upon in future studies.

## Author contributions

JT designed the experiments; AS and JT performed data analysis; AS and JT wrote the manuscript.

### Conflict of interest statement

The authors declare that the research was conducted in the absence of any commercial or financial relationships that could be construed as a potential conflict of interest.
